# Immune Checkpoint Inhibitor Toxicity in Head and Neck Cancer: From Identification to Management

**DOI:** 10.3389/fphar.2019.01254

**Published:** 2019-10-23

**Authors:** Haiyang Wang, Abdulkadir Mustafa, Shixi Liu, Jun Liu, Dan Lv, Hui Yang, Jian Zou

**Affiliations:** Department of Otolaryngology Head and Neck Surgery, West China Hospital, Sichuan University, Chengdu, China

**Keywords:** head and neck cancer, immunotherapy, immune checkpoint inhibitors, immune-related adverse events, programmed cell death 1, programmed death-ligand 1, cytotoxic T-lymphocyte-associated antigen-4

## Abstract

Benefiting from the continuously clarifying underlying biology of immune checkpoints and ligand–receptor interactions, the emergence of new anticancer treatment strategy, immunotherapy has shown substantial benefits on several liquid and solid tumors. Immune checkpoint inhibitors (ICIs) can block the negative regulatory components and enhance the T cell function, thus leading to prominent anticancer activity. On account of their promising effect on various malignancies shown in clinical trials, ICIs have been considered to be the most potent anticancer agents in the near future. Head and neck cancer is the seventh most common neoplasm worldwide, and the gross 5-year survival rate was only 60%. Managing locoregionally advanced, recurrent, or metastatic head and neck tumors is still a challenging problem for both oncologists and surgeons. Recent clinical trials employing the immune-modulating antibodies that target cytotoxic T-lymphocyte-associated antigen-4 (CTLA-4) and programmed cell death 1 (PD-1) herald a new era of anticancer therapy. However, like all other anticancer drugs, ICIs also have side effects while upregulating the immune system to enhance antitumor response, which were known as immune-related adverse events (irAEs). Generally, most irAEs were transient, but sometimes they can cause serious organ dysfunction, even fatal. In addition, due to the distinct anatomical feature, advanced head and neck tumors often affect the upper aerodigestive tract and cause serious dyspnea or dysphagia. Toxicities of ICIs may be more lethal for such patients. Thus, with the increasing application of anti-checkpoint agents in head and neck cancer, there is urgent need to ascertain the safety of this novel treatment strategy. Here, we compile this review of existing clinical trials on the toxicity of ICIs during cancer treatment. The particular clinical manifestation, characteristics of complication development in fatal cases, and the management strategies were discussed. This may provide vital information for future oncology trials and clinical practice.

## Introduction

Head and neck cancer is the seventh most common neoplasm worldwide, and the gross 5-year survival rate was only 60% (Torre et al., 2015). Managing locoregionally advanced, recurrent, or metastatic head and neck tumors is still a challenging problem for both oncologists and surgeons. In recent years, the development of immune checkpoint inhibitors (ICIs) has demonstrated a significant anticancer activity in different types of malignancies, including head and neck cancer. Treatment with ICIs improved the overall survival in patients with head and neck squamous cell carcinoma (HNSCC) and improved quality of life compared with the concurrent chemotherapy and radiation therapy (Porceddu and Haddad, 2017; Ribas and Wolchok, 2018).

Physiologically, immune checkpoint proteins are responsible for regulating immune tolerance and avoiding excessive immune injury. One of the main causes of the recurrence and metastasis of HNSCC is tumor-induced immune evasion, which is partially mediated by T cell-suppressive immune checkpoint (Ferris, 2015). ICIs facilitate endogenous anticancer activity by removing the inhibition signals and enhancing the activity of T cells. Currently, cytotoxic T-lymphocyte-associated antigen 4 (CTLA-4) and programmed cell death 1 (PD-1) are the ICIs that cause the most clinical interest. CTLA-4 is expressed in activated CD8^+^ T cells and is involved in the regulation of the early state of T cell activation. Furthermore, CTLA-4 mainly provides significant negative signals to inhibit the activation of T cells and weaken the anti-tumor immune response. PD-1 can be expressed on both the T and B cell’s surface. When PD-1 binds to its ligands PD-L1 or PD-L2, it releases inhibitory signals to T cells and decreases the downstream signal transmission through the PI3K pathway, resulting in the inhibited activation and proliferation of T cells (Stambrook et al., 2017; Szturz and Vermorken, 2017).

Nivolumab (an anti PD-1 agent) was the first ICI approved by the FDA for HNSCC therapy in 2016, based on results from the CheckMate141 and KEYNOTE-012 trial (Alfieri et al., 2018; Yang et al., 2018). Currently, the ICIs that have been tested on HNSCC include PD-1 (nivolumab and pembrolizumab), PD-L1 (atezolizumab, durvalumab, and avelumab), and CTLA-4 (ipilimumab and tremelimumab) (Alsaab et al., 2017; Szturz and Vermorken, 2017; Dogan et al., 2018; Gong et al., 2018).

Despite their therapeutic promise and benefits, treatments with ICIs are associated with the onset of immune-related adverse events (irAEs) on account of facilitating autoimmune activity not only against tumor cells but also any organs of the body. In fact, during HNSCC treatment, ICIs may lead to significant morbidity or, in rare cases, mortality. Identification of the side effects and prompt treatment are crucial for patients receiving these agents (Saba et al., 2017; Pauken et al., 2019). Based on all the existing eight clinical trials of ICI use in HNSCC, here, we conducted this review and summarized all the irAEs, the fatal complications, and the management strategies.

## Clinical Characteristics of Iraes and Management—General Principles

After a comprehensive retrieval of online databases including Pubmed, ISI, and clinicaltrials.gov, there were eight clinical trials that use ICI agents to treat HNSCC. General information on these trials is shown in **Table 1**. According to the eight trails, irAEs seem to most commonly involve the skin, gastrointestinal tract, endocrine glands, pulmonary and musculoskeletal. The incidence of irAEs (of any grade) among patients taking ICI in HNSCC ranges from 57% to 67%, and the most common irAEs of all grades were pruritus/rash, diarrhea, and hypothyroidism. More serious irAEs occur less frequently; grades 3–4 occur in 8–17% of patients treated with ICI agents. A list of the current ICIs and their common associated irAEs in HNSCC is shown in **Table 2**.

**Table 1:** Overview of the included clinical trials of ICIs in HNSCC.

NCT number	Study type	No. of patients	ICI dose	RR (%)	Median OS (months)
02105636 (Checkmate-141)	Randomized phase III	240	Nivolumab 3 mg/kg, every 2 weeks	13.3	7.5
01848834 (Keynote-012)	Phase Ib	192	Pembrolizumab 10 mg/kg, every 2 weeks OR Pembrolizumab 200 mg every 3 weeks	18	8
02252042 (Keynote-040)	Randomized phase III	247	Pembrolizumab 200 mg every 3 weeks	14.6	8.4
02255097 (Keynote-055)	Phase II	171	Pembrolizumab 200 mg every 3 weeks	16	8
01375842	Phase Ia	32	Atezolizumab 15/20 mg/kg every 3 weeks	21	6
01693562	Phase I/II	62	Durvalumab 10 mg/kg every 2 weeks	6.5	8.4
02207530	phase II	112	Durvalumab 10 mg/kg every 2 weeks	16.2	7.1
02319044	Randomized phase II	133	Durvalumab (20 mg/kg every 4 weeks) + tremelimumab (1 mg/kg every 4 weeks)	7.8	7.6
		67	Durvalumab (10 mg/kg every 2 weeks)	9.2	6
		67	Tremelimumab (10 mg/kg every 4 weeks	1.6	5.5

NCT, national clinical trial; ICIs, immune checkpoint inhibitors; RR, response rate; OS, overall survival; HNSCC, head and neck squamous cell carcinoma.

**Table 2:** List of current ICIs and their associated common toxicities in HNSCC therapy.

Drug class	Drug name	No. of trials mentioned	Adverse events (%)
PD-1 inhibitors	Nivolumab	*n* = 1 (Ferris et al., 2018)	Dermatological (15.7%), Hypothyroidism (6.3%), Diarrhea (6.8%)
	Pembrolizumab	*n* = 4 (Chow et al., 2016; Seiwert et al., 2016; Bauml et al., 2017; Cohen et al., 2019)	Hypothyroidism (9–16%), Dermatological (8–19%), Diarrhea (6–8%)
PD-L1 inhibitors	Atezolizumab	*n* = 1 (Colevas et al., 2018)	Dermatological (16%), Diarrhea (9%)
	Durvalumab	*n* = 3 (Siu et al., 2018; Segal et al., 2019; Zandberg et al., 2019)	Diarrhea (5.4–10.8%), Dermatological (6.3–13%), Hypothyroidism (3.2–10.8%)
CTLA-4 inhibitors	Tremelimumab	*n* = 1 (Siu et al., 2018)	Diarrhea (15.4%), Dermatological (12.3%)
PD-L1 + CTLA-4 inhibitors	Durvalumab + Tremelimumab	*n* = 1 (Siu et al., 2018)	Diarrhea (14.3%), Hypothyroidism (8.3%)

PD-1, programmed death receptor-1; PD-L1, programmed death-ligand 1; CTLA-4, cytotoxic T-lymphocyte-associated protein 4; ICIs, immune checkpoint inhibitors; HNSCC, head and neck squamous cell carcinoma.

In general, management of moderate to severe irAEs relies on the use of corticosteroids, other immunomodulatory agents, supportive care, and treatment interruption (Spain et al., 2016; Haanen et al., 2017; Puzanov et al., 2017; Brahmer et al., 2018; Kottschade, 2018; Williams et al., 2019). The Common Terminology Criteria for Adverse Events (CTCAE) is the standard assessment used in clinical trials to grade the severity of adverse events. Generally, grade 1–2 irAEs are mild to moderate, do not require hospitalization, and should be treated symptomatically. Grade 3–4 irAEs are severe to life-threatening conditions, which require hospitalization to observe patients closely. Systemic steroid administration and permanent discontinuation of ICI therapy may be required. Grade 5 refers to death related to adverse events.

## Common IRAES and the Relevant Management in HNSCC

### Dermatological Adverse Events

Dermatologic toxicities are one of the most reported irAEs associated with immune checkpoint inhibitors and occur in 8–19% of HNSCC patients treated with ICI agents (Chow et al., 2016; Seiwert et al., 2016; Bauml et al., 2017; Colevas et al., 2018; Ferris et al., 2018; Siu et al., 2018; Cohen et al., 2019; Segal et al., 2019; Zandberg et al., 2019). The majority of cases are usually low grade, ranging from pruritus and rash to dermatitis. However, serious skin reaction is less common (grades 3–4 are less than 2%). Cases of Stevens–Johnson syndrome are practically notable, and one case resulted in a treatment-related death, with pembrolizumab treatment (Cohen et al., 2019).

Skin biopsy is useful to rule out any other etiologies, such as an infection and drug interaction or other autoimmune blistering dermatoses. Grade 1–2 rashes (macules/papules covering less than 10% or 10–30% of body surface area) can be treated with topical emollients, oral anti-histamine, and topical steroids; consider initiating prednisone 0.5–1 mg/kg in grade 2 skin reaction, with tapering over at least for 4 weeks. For grade 3 skin reaction (> 30% BSA), withhold the ICI therapy and manage with systemic high-dose steroid prednisone 1–2 mg/kg (or equivalent), with tapering over at least for 4 weeks. For grade 4 (life-threatening) skin reactions such as Stevens–Johnson syndrome/toxic epidermal necrolysis, treatment consists of permanent discontinuation of ICI therapy, supportive care, and intravenous steroid. Intravenous immunoglobulin or cyclosporine is considered in severe or corticosteroid-unresponsive cases.

### Endocrine Adverse Events

Endocrine toxicity is a common side effect of ICI therapy in HNSCC patients. The most frequent endocrinopathies are hypothyroidism, hyperthyroidism, and hypophysitis. Rare cases of primary adrenal insufficiency, hypercalcemia, and immune-related type 1 diabetes mellitus, leading to hyperglycemia and diabetic ketoacidosis, have been reported (Chow et al., 2016; Seiwert et al., 2016; Bauml et al., 2017; Colevas et al., 2018; Ferris et al., 2018; Siu et al., 2018; Cohen et al., 2019; Segal et al., 2019; Zandberg et al., 2019). Routine monitoring of thyroid function tests, hormone levels testing, and glucose level before starting therapy and before each dose are required. Consultation with an endocrinologist is recommended in all cases of suspected endocrinopathies.

Hypothyroidism is the most common endocrinopathy and occurs in higher incidence with anti-PD-1/PD-L1 agents in HNSCC patients (6.8–16%), and the majority of cases are with mild symptoms. For patients treated with tremelimumab (CTLA-4 inhibitors), the incidence rate of hypothyroidism is less than 2% (Siu et al., 2018).

Typically, hypothyroidism [elevated thyroid-stimulating hormone (TSH), normal or low FT4] presents with nonspecific symptoms such as fatigue, asthenia, cold intolerance, and dry skin. The management of hypothyroidism consists of thyroid hormone replacement (levothyroxine), usually starting with 0.8–1.0 µg kg^−1^ day^−1^ (Ma et al., 2019) and supportive care. ICI therapy is withheld in severe cases until symptoms resolve to baseline with appropriate supplementation.

Hyperthyroidism was reported with low frequency (about 3%) and may present with a new onset of atrial fibrillation, heat intolerance, and weight loss. Cardiovascular and neurological symptoms are relieved by a beta-blocker (atenolol 25–50 mg/day) and supportive care. In severe cases, the ICI therapy is withheld until symptoms resolve to baseline. In the most recent study of MA and colleagues, high-dose glucocorticoids (HDGs) did not improve the outcome of ICI-related thyroid disorders; therefore, routine use of HDGs in patients with suspected thyroid disorders such as thyrotoxicosis is not recommended. In addition, they suggested using HDGs in patients who present with symptoms of thyroid storm or in patients with cardiac disease (Ma et al., 2019).

The incidence of hypophysitis in HNSCC patients treated with ICI agents is less than 1%. Hypophysitis patients will present with headache, fatigue, and with multiple hormone deficiencies [adenocorticotropic hormone (ACTH), TSH, follicle-stimulating hormone, luteinizing hormone, growth hormone, and prolactin]. The diagnosis of hypophysitis is confirmed with pituitary magnetic resonance imaging, which shows the enlargement of the pituitary and thickening of the stalk. Laboratory studies distinguish hypophysitis from primary adrenal insufficiency (low cortisol and high ACTH) and primary hypothyroidism (high TSH and low FT4). Tissue biopsy is the definitive diagnosis for lymphocytic hypophysitis. Treatment consists of long-term hormone replacement with supportive care and withholding ICI therapy. In severe symptoms, treatment is with the high-dose corticosteroid prednisone 1–2 mg/kg (or equivalent), with tapering over at least for 4 weeks.

### Gastrointestinal Adverse Events

Diarrhea (an increase in the frequency of stools) is one of the more frequently observed irAEs with ICIs, and the majority of cases are mild. The incidence of diarrhea/colitis is higher in patients receiving the durvalumab + tremelimumab combination arm (14.3%) or in the tremelimumab arm (16.9%), whereas patients treated with anti PD-L1 alone experience less frequent cases (5.4–10.4%) (Siu et al., 2018). Moreover, the incidence of all grade diarrheas is lower with anti PD-1 drugs, less than 8% (Chow et al., 2016; Seiwert et al., 2016; Bauml et al., 2017; Ferris et al., 2018; Cohen et al., 2019). This gave us an implication that HNSCC patients are more prone to develop diarrhea during CTLA-4 therapy.

Symptoms of colitis are diarrhea accompanied with abdominal pain and, occasionally, rectal bleeding. Severe colitis can be a life-threatening condition and result in intestinal perforation. The upper gastrointestinal (GI) tract is less commonly affected. Symptoms such as dysphagia and epigastric pain have been reported (Chow et al., 2016; Seiwert et al., 2016; Bauml et al., 2017; Siu et al., 2018; Cohen et al., 2019; Zandberg et al., 2019).

A stool analysis, including bacterial cultures to exclude other etiologies such as infections with *Clostridium difficile* or other bacterial or viral pathogens, is required. Colonoscopy or sigmoidoscopy and abdominal CT scan are helpful in patients with bloody diarrhea or severe diarrhea (≥7 stool per day over baseline). Upper endoscopy is indicated in patients with upper GI symptoms.

Grade 1–2 diarrhea is managed with supportive care and antidiarrheal medication. Patients whose diarrhea progresses and/or is grade 3 or higher should withhold ICI therapy and treat with prednisone 1 mg/kg or equivalent, with tapering over at least for 4 weeks. In severe or life-threatening enterocolitis, the ICI therapy should be discontinued permanently and a high dose of corticosteroids given, prednisone 1–2 mg/kg (or equivalent), with tapering over for 4–6 weeks. In addition, infliximab (anti-TNFα monoclonal antibody) 5–10 mg/kg is recommended for patients not improved with steroids or to use mycophenolate mofetil if infliximab cannot be used.

### Hepatic Adverse Events

Hepatitis is observed in 1–8% of HNSCC patients treated with immune checkpoint inhibitors, with severe adverse events (grades 3–5) occurring in 1–3%. Most patients present with asymptomatic elevation of aspartate aminotransferase and alanine transferase, but may present with hyperbilirubinemia, jaundice, and fatigue in advanced cases.

Liver function tests, liver enzyme test, and viral hepatitis serology are recommended prior to initiating ICI therapy. Biopsy and radiological tests could be considered to rule out other etiologies. Imaging may be helpful to rule out disease progression. Management consists of withholding ICI administration and prompt treatment with corticosteroid for moderate cases. For grade 3 or higher, treat with a high dose of corticosteroid, prednisone 1–2 mg/kg (or equivalent), with tapering over at least for 4 weeks, and ICI therapy should be permanently discontinued if there is no improvement with corticosteroids and liver function still elevated. In addition, mycophenolate mofetil IV 1 g twice a day is suggested for cases refractory to steroids. However, infliximab not given to patients with elevated AST/ALT since infliximab can cause hepatic injury.

### Pulmonary Adverse Events

Pneumonitis is a noninfectious inflammation of the lung which occurs in 1–4% of HNSCC patients receiving ICI therapy. This condition can be severe and life-threatening. The most common symptom of pneumonitis is dyspnea (shortness of breath), which may be accompanied by dry cough and hypoxia. The incidence of pneumonitis is lower in HNSCC patients compared to patients with non-small cell lung cancer (NSCLC) (Suresh et al., 2018).

In general, lung biopsy and bronchoscopy might be indicated to exclude infection and other causes. Patients with suspected pneumonitis should undergo a CT scan. Imaging usually shows interstitial infiltrates and ground-glass opacities. In a recent study, Colen et al. reported several radiomic features that can be used to predict patients at risk for immunotherapy-induced pneumonitis, such as the maximum relevance and minimum redundancy feature selection method, anomaly detection algorithm, and leave-one-out cross-validation which identified radiomic features that were significantly different (Colen et al., 2018).

The American Society of Clinical Oncology (ASCO) guideline recommends withholding ICI therapy for any grade pneumonitis (Brahmer et al., 2018). For grade 2 pneumonitis, treat with systemic steroids, prednisone 1–2 mg kg^−1^ day^−1^ (or equivalent), and empirical antibiotics in the case of infections and withhold ICI therapy. Grade 3 or higher pneumonitis treatment consists of permanently discontinuing the use of ICI therapy and using a high dose of intravenous corticosteroids, (methyl)prednisone 1–2 mg kg^−1^ day^−1^ with additional immunosuppression (infliximab 5 mg/kg or mycophenolate mofetil IV 1 g twice a day or IVIG for 5 days or cyclophosphamide). Steroids should be tapered slowly over weeks.

### Rheumatologic/Musculoskeletal Adverse Events

Musculoskeletal side effects are commonly seen with the ICI therapy trials. Arthralgia and myalgia are the most common and occur in 2–6% percent in the HNSCC patients treated with ICI agents (Chow et al., 2016; Seiwert et al., 2016; Bauml et al., 2017; Colevas et al., 2018; Siu et al., 2018; Segal et al., 2019; Zandberg et al., 2019). The incidence of grade 3 or higher was rare with musculoskeletal irAEs. One case of grade 3 musculoskeletal pain had been reported with pembrolizumab therapy (Seiwert et al., 2016).

Early rheumatologic consultation is advised. The diagnostic workup should include complete rheumatologic and neurological history and examination including muscle strength. Autoimmune blood panel and inflammatory markers such as erythrocyte sedimentation rate and C-reactive protein may be considered for inflammatory arthritis (Brahmer et al., 2018).

Manage arthralgia and myalgia with analgesia with paracetamol and/or NSAIDS. For moderate symptoms, treat with low-dose prednisolone 10–20 mg/day or equivalent for 4–6 weeks and withhold ICI therapy. For severe symptoms, withhold ICI therapy and treat with high-dose corticosteroid prednisone 1–2 mg/kg (or equivalent). In severe arthritis, anti-TNFα therapy should be initiated. Plasmapheresis and intravenous immunoglobulin therapy or other immunosuppressant therapy may be considered for severe myositis cases.

## Less Common IRAES and the Relevant Management in HNSCC

Besides the common irAEs mentioned above, ICI toxicity can also affect other body organs, including the neurologic, cardiovascular, and renal systems, based on the existing clinical trials. The incidence of these adverse effects may be relatively low, but sometimes the consequences can be extremely serious and even lethal (**Table 3**).

**Table 3:** List of uncommon iRAEs of ICIs in HNSCC therapy.

Organs	Disease	Drug classes	Incidence of grade 3/4 toxicity
Neurologic	Guillain–Barré syndrome	PD-1	≤1%
Cardiovascular	Congestive cardiac failure, atrial fibrillation, and cardiac tamponade	PD1 and PD-L1	2–3%
Renal	Nephritis	PD-L1	≤1%

PD-1 programmed death receptor-1; PD-L1 programmed death-ligand 1; ICIs immune checkpoint inhibitors; HNSCC head and neck squamous cell carcinoma; iRAEs immune-related adverse events.

Neurologic irAEs remain an uncommon toxicity, and the incidence of grade 3 or higher is less than 1% in HNSCCs. A range of neurologic events have been described, which include Guillain–Barré syndrome, encephalitis, and peripheral neuropathy. One case of severe adverse event (grades 3–5), Guillain–Barré syndrome, has been reported with pembrolizumab (Cohen et al., 2019). Diagnosis of neurologic toxicity comes from nerve conduction studies, lumbar puncture, and spine/brain MRI. Significant neurological toxicity should be managed with high-dose steroid and withholding the ICI therapy. For progressive Guillain–Barré syndrome, intravenous immunoglobulin (0.4 g kg^−1^ day^−1^ for 5 days) or plasmapheresis should be initiated. Frequent neurologic evaluation and pulmonary function monitoring are recommended. Patients with peripheral neuropathy may be offered low-dose prednisolone 0.5–1 mg/kg, GABA agonist (e.g., pregabalin or duloxetine) and withhold the ICI therapy. Patients with aseptic meningitis or encephalitis are managed with methylprednisolone 1–2 mg/kg and empiric antiviral (IV acyclovir).

Cardiovascular irAEs are uncommon and seen in less than 3% of HNSCC patients on ICI therapy and can be associated with general myositis. Several cases of cardiac tamponade, arrhythmias, and congestive heart failure were reported (Chow et al., 2016; Seiwert et al., 2016; Bauml et al., 2017; Colevas et al., 2018; Siu et al., 2018; Cohen et al., 2019; Segal et al., 2019; Zandberg et al., 2019). Diagnosis of cardiac toxicity can be established by electrocardiogram, cardiac biomarkers, and cardiac magnetic resonance imaging. Early consultation with a cardiologist is recommended. Management consists of a high dose of intravenous corticosteroids, prednisone 1–2 mg kg^−1^ day^−1^, with additional immunosuppression (e.g., infliximab 5 mg/kg, intravenous immunoglobulin or mycophenolate mofetil) and withholding the ICI therapy.

Renal irAEs are less common and occur in less than 3% of patients on ICI therapy. One case of grade 3 nephritis has been reported with durvalumab therapy (Segal et al., 2019). Serum creatinine should be monitored prior to starting therapy and before each dose. Renal biopsy can be considered to rule out other causes. For moderate nephritis cases, withhold ICI therapy and treat with prednisone 0.5–1 mg/kg or equivalent, with tapering over at least for 6 weeks. In severe or life-threatening nephritis, the ICI therapy should be discontinued permanently and a high dose of corticosteroids is given, prednisone 1–2 mg/kg or equivalent, with tapering over for 4–6 weeks. In addition, dialysis may be required for patients with severe renal failure.

## IRAE-Related Death in HNSCC

The overall incidence of irAE-related death is low, but does occur at a rate of 0.3% to 1.03% (Wang et al., 2018). In a recent review by Jiang et al., the most common CTLA-4 treatment-related death was gastrointestinal toxicity, and the most PD-1 treatment-related death was pulmonary toxicity (Jiang et al., 2019). In our analysis, we observed six fatal irAEs in HNSCC. Three deaths were reported in patients treated with pembrolizumab and two deaths were reported with nivolumab therapy; one death was reported with combination therapy (durvalumab + tremelimumab). Detailed information was listed in **Table 4**.

**Table 4:** Baseline characteristics of death cases and involved clinical trials.

Involved organs	Treatment-related death	NCT no.	ICI	Dose of ICI
**Pulmonary (** ***n*** **= 3)**	Pneumonitis (*n* = 2)	02105636 (*n* = 1)	Nivolumab	3 mg/kg every 2 weeks
		02255097 (*n* = 1)	Pembrolizumab	200 mg every 3 weeks
	ARF (*n* = 1)	02319044 (*n* = 1)	Durvalumab + Tremelimumab	Durvalumab (20 mg/kg every 4 weeks) + tremelimumab (1 mg/kg every 4 weeks)
**Dermatological (** ***n*** **= 1)**	SJS (*n* = 1)	02252042 (*n* = 1)	Pembrolizumab	200 mg every 3 weeks intravenously
**Gastrointestinal (** ***n*** **= 1)**	LIP (*n* = 1)	02252042 (*n* = 1)	Pembrolizumab	200 mg every 3 weeks
**Endocrine (** ***n*** **= 1)**	Hypercalcemia (*n* = 1)	02105636 (*n* = 1)	Nivolumab	3 mg/kg every 2 weeks

NCT, national clinical trial; ICI, immune checkpoint inhibitor; ARF, acute respiratory failure; SJS, Stevens–Johnson syndrome; LIP, large intestine perforation.

Two patients died of treatment-related pneumonitis: one in the CheckMate-141 trial and another in the single-arm phase II KEYNOTE-055 trial (Bauml et al., 2017; Ferris et al., 2018). Pneumonitis is less common irAEs, but it is one of the most common causes of ICI-related deaths. In addition, the incidence of treatment-related death is higher with anti-PD-1 therapy and typically occurs later than other irAEs. In a phase II/III study on the efficacy and safety of pembrolizumab in patients with advanced NSCLC, three cases of pneumonitis-related deaths (3 of 682 NSCLC patients) were reported in Herbst’s study (Herbst et al., 2016).

Treatment-related death, Stevens–Johnson syndrome (SJS), occurred in one patient treated with pembrolizumab in the KEYNOTE-040 trial (Cohen et al., 2019). SJS is a severe life-threatening cutaneous adverse reaction, which is, in most cases, drug-induced. Patients present with purpuric rashes with blisters, oral mucositis, and conjunctivitis. SJS involves <10% body surface area skin detachment. Super infection, massive fluid losses, and electrolyte imbalances can lead to death (Harr and French, 2010; Plachouri et al., 2019; Woolum et al., 2019).

Incidence of anti-PD-1/PD-L1 perforating colitis is less frequent compared to anti-CTLA-4 treatment-associated perforated colitis. However, treatment-related death occurred in one patient treated with pembrolizumab (large intestine perforation induced by colitis) in the KEYNOTE-040 trial (Cohen et al., 2019).

One patient died from treatment-related acute respiratory failure in the combination therapy arm (durvalumab + tremelimumab) in the phase II CONDOR randomized clinical trial. The primary cause of death was squamous cell carcinoma disease progression (Siu et al., 2018). In the CheckMate 141 Study, one patient in the nivolumab group died from treatment-related hypercalcemia (Ferris et al., 2018).

The risk of fatal irAEs is very low and typically occurs in the early phase during treatment. It is vital for the clinician to be aware of these potential lethal complications. Early recognition, proper intervention, and long-term monitoring of potential fatal adverse events may be effective in preventing treatment-related death.

## Future Perspective and Ongoing Clinical Trials for the Treatment of HNSCC

**Figure 1:**
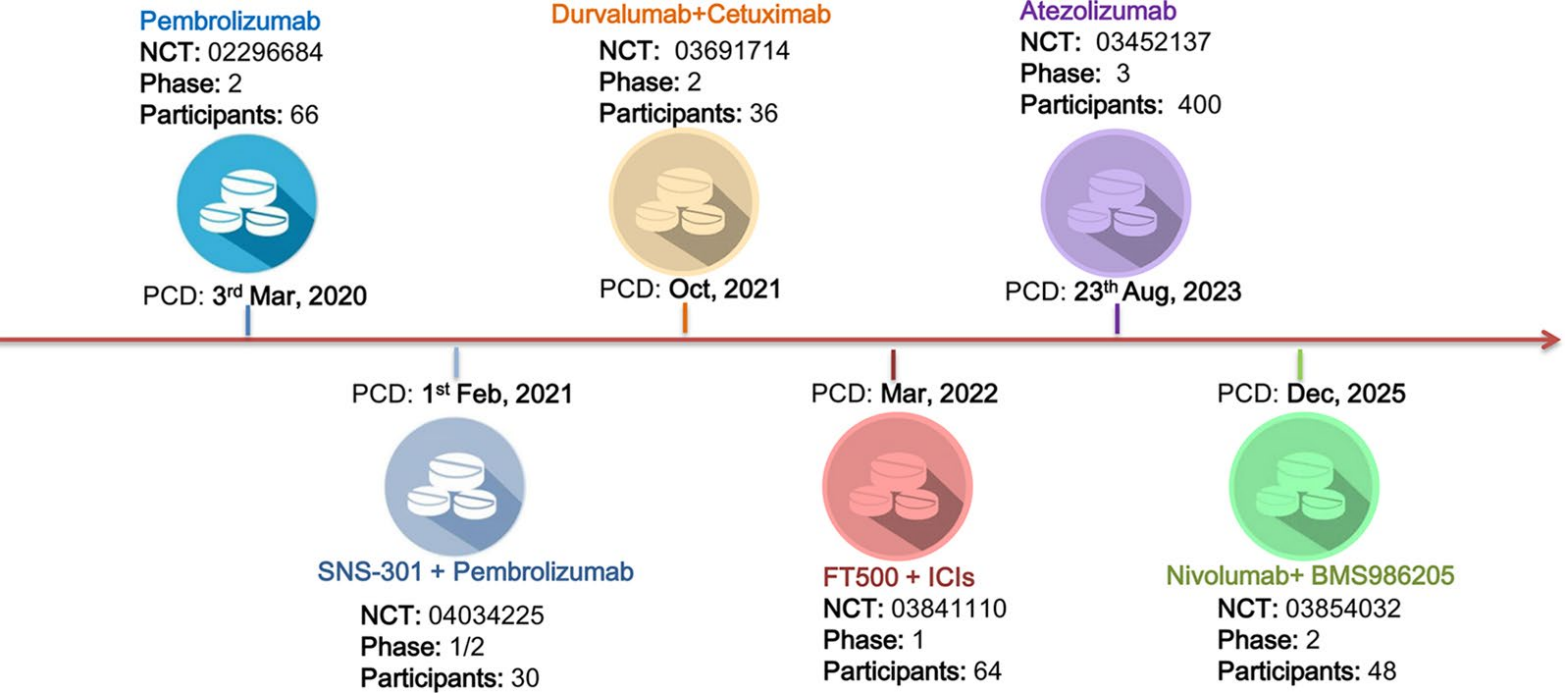
Ongoing clinical trials for the treatment of head and neck squamous cell carcinoma (HNSCC). There were six registered ongoing trials as of August 2019. Four of them were designed in combining intervention strategy. The other two were undergoing phase 2 and phase 3 trials for Pembrolizumab and Atezolizumab in HNSCC therapy, respectively. *PCD* primary completion date.

Although ICIs have been approved by the FDA as the second-line treatment of recurrent and/or metastatic HNSCC, the relatively high rate of irAEs and the low response rate call for new immunotherapy strategies, either in monotherapy or in combination with existing ICIs. As of August 2019, there are six ongoing clinical trials evaluating ICIs in HNSCC (**Figure 1**). Pembrolizumab and Atezolizumab are now undergoing phase 2 and phase 3 trials. Notably, the remaining four trials were designed as ICIs in combination with other agents, including SNS-301, Cetuximab, FT500, and BMS986205.

SNS-301 is a cancer vaccine that targets human aspartate β-hydroxylase (ASPH), which had been found overexpressed in many solid tumors, but would not be expressed in human after fetal development. A phase I study in prostate cancer had confirmed the safety and tolerability of SNS-301. The ASPH-specific immune activity had also been established in this study, which provided the foundation for phase II study in solid and hematological tumors. Cetuximab is a well-known anti-EGFR monoclonal antibody that has been widely used in HNSCC. A recent study published by Andre et al. suggested that cetuximab plus monalizumab, a humanized anti-NKG2A antibody, had a promising response rate (31%) and common irAEs (ranging from 10% to 17%) compared to current ICIs in HNSCC therapy. The involved mechanism had also been suggested as monalizumab can enhance natural killer (NK) cell activity against tumor cells and rescue CD8^+^ T cell function (Andre et al., 2018). FT500, a NK cell product derived from the clonal master iPSC line, may overcome the multiple mechanisms of ICI resistance, including recognition and lysis of tumor cells upon the downregulation of HLA-1 on tumor cells. In AACR 2018, Bjordahl and his colleagues reported that FT500 can facilitate the T cell recruitment and enhance T cell activation (Bjordahl et al., 2018). BMS986205 is a novel enzymatic-targeted drug that belongs to the indolamine-2,3-dioxygenase-1 (IDO-1) inhibitors, which can restore the differentiation of T cells and downregulate the immunosuppressive effect of kynurenine. Combining IDO-1 inhibitors with anti-PD1/PD-L1 was shown effective in many kinds of solid tumors. In a phase 1/2a trial, the treatment-related adverse events range from 6.8% to 18.2%, and there were no grade 4 or 5 adverse events (Zhu et al., 2019).

The irAEs and the response rates differing from various tumor types are the main challenges for the first-generation immunotherapy. Fortunately, the safety and efficiency of ICIs in different tumors are now getting more distinct. Moreover, combining ICIs with other agents, including but not limited to anti-ASPH vaccine, anti-EGFR monoclonal antibody, IDO-1 inhibitors, and NK cell products, may shed light to complement current HNSCC immunotherapy.

## Conclusions

Overall, our results did not show higher rates or severity of irAEs in HNSCC patients compared with other malignancies. The most common reported adverse events are dermatologic, endocrine, and gastrointestinal. Higher rates of endocrine disorders are associated with anti-PD-1 therapy, whereas gastrointestinal toxicities are more common with CTLA-4 inhibitor administration. In addition, pneumonitis seems to be associated with a higher risk of ICI treatment-related death in HNSCC. This raised a claim that clinicians must maintain sharp vigilance on the respiratory symptoms and early intervention should be taken once the pneumonitis was suspected.

General management of irAEs includes treatment interruption, immunosuppression or immunomodulatory agents, and hormone replacement. Glucocorticoid therapy was commonly used for moderate to severe irAEs. But it must be noted that routine use of HDGs may not be suitable for all adverse events, including but not limited to ICI-related thyroid disorders.

As the use of immunotherapy increases, patients receiving ICI therapy are at risk of developing irAEs that may lead to severe or fatal toxicities. Knowledge of these adverse events and the management algorithm discussed in this paper will provide an important tool for clinicians and for future oncology trials.

## Author Contributions

The original idea of this study was from HW. HW and AM reviewed the literature and contributed equally to the manuscript writing and editing. JZ instructed the whole manuscript formation. SL and JL contributed to the literature retrieval and review. DL and HY contributed to the manuscript editing and proofreading.

## Funding

This work was supported by the Sichuan Science and Technology Program under grant no. 2017JY0253 and Major programs of Sichuan Science and Technology Department no. 2017SZ0015.

## Conflict of Interest

The authors declare that the research was conducted in the absence of any commercial or financial relationships that could be construed as a potential conflict of interest.
